# The Effect of Lithium Doping on the Sintering and Grain Growth of SPS-Processed, Non-Stoichiometric Magnesium Aluminate Spinel

**DOI:** 10.3390/ma9060481

**Published:** 2016-06-16

**Authors:** Yuval Mordekovitz, Lee Shelly, Mahdi Halabi, Sergey Kalabukhov, Shmuel Hayun

**Affiliations:** Department of Materials Engineering, Ben-Gurion University of the Negev, P.O. Box 653, Beer-Sheva 8410501, Israel; yuvalmor@post.bgu.ac.il (Y.M.); leeshel@post.bgu.ac.il (L.S.); mahdi@post.bgu.ac.il (M.H.); kalabukh@bgu.ac.il (S.K.)

**Keywords:** grain growth, lithium, magnesium aluminate spinel, precipitation, SPS

## Abstract

The effects of lithium doping on the sintering and grain growth of non-stoichiometric nano-sized magnesium aluminate spinel were studied using a spark plasma sintering (SPS) apparatus. Li-doped nano-MgO·*n*Al_2_O_3_ spinel (*n* = 1.06 and 1.21) powders containing 0, 0.20, 0.50 or 1.00 at. % Li were synthesized by the solution combustion method and dense specimens were processed using a SPS apparatus at 1200 °C and under an applied pressure of 150 MPa. The SPS-processed samples showed mutual dependency on the lithium concentration and the alumina-to-magnesia ratio. For example, the density and hardness values of near-stoichiometry samples (*n* = 1.06) showed an incline up to 0.51 at. % Li, while in the alumina rich samples (*n* = 1.21), these values remained constant up to 0.53 at. % Li. Studying grain growth revealed that in the Li-MgO·*n*Al_2_O_3_ system, grain growth is limited by Zener pining. The activation energies of undoped, 0.2 and 0.53 at. % Li-MgO·1.21Al_2_O_3_ samples were 288 ± 40, 670 ± 45 and 543 ± 40 kJ·mol^−1^, respectively.

## 1. Introduction

Magnesium aluminate spinel (MgO·*n*Al_2_O_3_) is an attractive ceramic material for many technological applications, owing to its combination of excellent mechanical and optical properties [1,2,3]. To realize and maximize its qualities, the spinel must be sintered to full density. Sintering to full density is usually a difficult goal to achieve, given the requirements of high pressure and elevated temperatures. Yet, even then, variations in powder quality and densification processes can cause optical defects [4,5,6]. To overcome these issues, the use of sintering additives, such as Na_3_AlF_6_ [7], AlF_3_ [3], B_2_O_3_ [8], AlCl_3_ [3], CaO [2], LiF [9,10,11] and CaCO_3_ + LiF [4], has been proposed. Of these, it was established that LiF consistently allowed for the sintering of transparent spinel [6,9,10,11,12]. As such, the effect of LiF on the sintering behavior of MgAl_2_O_4_ has been extensively studied [6,9,10,11,12,13,14], including by Meir *et al.* [10] and Rosenburg *et al.* [11].

Two mechanisms were proposed to explain the enhanced sintering kinetics and improved transparency attained by the sintered parts. The first involves the formation of a liquid phase (LiF, melting point (m.p.) ~847 °C) at relatively low temperature that wets the MgAl_2_O_4_ particles and likely aids densification by particle rearrangement and liquid-phase sintering. The second mechanism was proposed to act at higher temperatures. Here, LiF decomposes and the highly reactive F^−^ ions react with impurities (e.g., C and S), thereby cleaning/activating particle surfaces. In turn, the Li^+^ cations react with the spinel, resulting in accelerated mass transport due to the formation of oxygen vacancies. Recently, we studied the effects of lithium on the energetics, thermal stability, and coarsening of MgO·*n*Al_2_O_3_, as well as its solubility in two-alumina-rich spinel compositions (*n* = 1.06 and *n* = 1.21). It was established that the phase stability of Li-doped, near-stoichiometry (*n* = 1.06) spinels is size-dependent. The spinel structure was able to hold up to 1 at. % lithium at grain sizes smaller than 30 nm, whereas for larger crystallite sizes, Mg(Li,Al)O and γ-LiAlO_2_ phases precipitated. The aluminum-rich samples (*n* = 1.21) showed greater phase stability, with decomposition occurring only above 1 at. % lithium, independent of crystallite size. The measured surface (and interface) enthalpies of MgO·1.06Al_2_O_3_, MgO·1.21Al_2_O_3_ and 0.20 at. % Li-MgO·1.21Al_2_O_3_ were 1.51 ± 0.15 (0.42 ± 0.20) Jm^−2^, 1.17 ± 0.15 (0.32 ± 0.21) and 1.05 ± 0.12 (0.24 ± 0.18) Jm^−2^, respectively [15]. These values are in agreement with the lower coarsening tendency of aluminum-rich spinels [15]. Spark plasma sintering is a well-established method for sintering transparent magnesium aluminate spinel [10,16,17,18,19,20,21,22,23,24] which combines axial pressure with heating via an electrical current passing through a die containing the powder body. A LiF sintering additive (~1 wt. %) is typically required for transparency.

In the present work, dense bodies from various lithium-doped nano-MgO·1.06Al_2_O_3_ and MgO·1.21Al_2_O_3_ spinels were SPS-processed and their microstructure and phase composition were analyzed. The cardinal role of the Li additive is emphasized and discussed.

## 2. Materials and Experimental Procedures

Li-doped nano-MgO·*n*Al_2_O_3_ spinel (*n* = 1.06 and 1.21) powders containing 0, 0.20, 0.50 or 1.00 at. % Li were synthesized by the solution combustion method [25], as described in detail by Mordekovitz and Hayun [15]. A 100 mL water-based solution was prepared with the appropriate amount of magnesium nitrate (Mg(NO_3_)_2_·6H_2_O, 96% metal basis, Fluka Analytical, St. Louis, MO, USA), aluminum nitrate (Al(NO_3_)_3_·9H_2_O, 96% metal basis, Fluka Analytical) and lithium acetate (LiCH_3_CO_2_·2H_2_O, reagent grade, metal basis, Alfa Aesar, Haverhill, MA, USA). Thirty-seven grams of citric acid (ACS reagent ≥99.5) and 6 mL ethylene glycol (anhydrous, 99.8%, Sigma Aldrich, St. Louis, MO, USA) were added to the solution. The resulting mixtures were evaporated at 120 °C under agitation by magnetic stirring until high-viscosity foam-like colloids had formed. Finally, the dried gel precursor was calcined at 850 °C for 72 h to obtain a fine powder. Sintering was conducted in a Spark Plasma Sintering Machine (FCT Systems GmbH, Rauenstein, Germany) using a modified elevated pressure set-up capable of delivering uniaxial pressures greater than 500 MPa. Ten millimeter disks were sintered using a graphite die (20 mm outer diameter) with silicon carbide (SiC) plungers placed inside a conventional 20 mm graphite die-and-plunger set. All SPS experiments were conducted in a low vacuum (1.3 hPa), with a K-type control thermocouple in contact with the outer wall of the ø10 mm die. The sintering procedure was conducted at 1200 °C under 150–300 MPa of uniaxial pressure. The heating rate was 50 °C/min and the holding time at the highest temperature was 15 min. Grain growth heat treatments were performed in air for 8, 24 and 72 h at a temperature range of 1300–1450 °C. X-ray powder diffraction (XRD) was performed using a Rigaku RINT 2100 diffractometer with Cu Kα radiation (Tokyo, Japan). The operating parameters were 40 KV and 40 mA with a 2θ step of 0.02°. Cell parameters were calculated from the diffractions obtained using the MDI Jade 2010 software package (version 2.8.1, 2014, Materials Data, Livermore, CA, USA).

Microstructure was studied using high-resolution scanning electron microscopy (HRSEM, JEOL-7400F, Tokyo, Japan) and by transmission electron microscopy (TEM) using a JEOL 2100 (Tokyo, Japan) microscope equipped with a high-angle annular dark-field (HAADF) GATAN detector. Samples for scanning electron microscope (SEM) characterization were prepared using a standard metallographic procedure, finalized by polishing with a 1 μm diamond paste. Polished specimens were thermally etched at the same heat treatment temperature for 6 min.

TEM and STEM (scanning transmission electron microscope) samples were prepared from a copper-matrix composite with the spinel samples being embedded in the soft copper matrix, as described in detail by Halabi *et al.* [26] this technique was used in order to overcome charge-related issues encountered during the TEM work. The spinel samples were ground and mixed with pure copper powder (~10 μm). Disks 3 mm in diameter and 70 μm thick were pressed and sintered at 700 °C in an N_2_ atmosphere. The perforation stage was carried out using a Gatan Dimpler and Precision Ion Polishing System. Grain size was estimated using Thixomet software [27] for image analysis. The density of the specimens was measured by the Archimedes method (ASTM Standard B-311 [28]), while Vickers hardness was measured using a Buehler–Micromet 2100 hardness tester (2 kg load, ASTM Standard C-1327 [29]). The samples were polished to an optical level for transmission measurements at 500 and 1000 nm wavelengths (Spectrophotometer V-1100D, MRC, Holon, Israel).

## 3. Results and Discussion

### 3.1. Phase Composition

**Figure 1:**
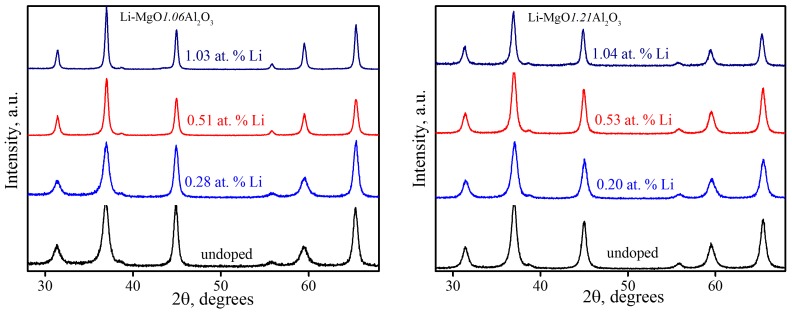
XRD patterns of as-synthesized powder samples.

Figure 1 shows XRD patterns for Li-doped and undoped nano-crystalline MgO·1.06Al_2_O_3_ and MgO·1.21Al_2_O_3_ samples synthesized by the combustion synthesis technique. The patterns indicate the presence of a spinel phase with relatively broad reflection peaks, suggesting small crystallite sizes calculated to range between 9.2 ± 0.2 and 32.5 ± 0.6 nm in the pure and doped samples (Table 1). Detailed characterization of the nano-powders prepared by this method can be found elsewhere [15].

**Figure 2:**
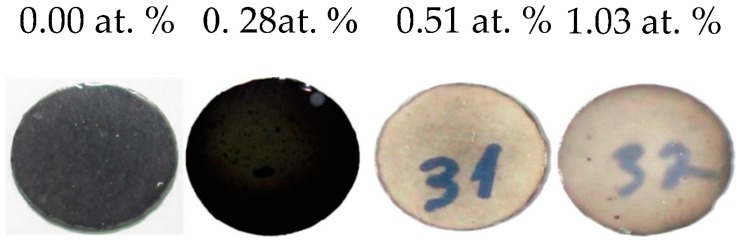
Photograph of Li-MgO·1.06Al_2_O_3_ SPS-processed samples. The polished specimens are 10 mm in diameter and ~1.5 mm thick. The effect of lithium on transparency is visible.

Typical SPS-processed specimens from as-synthesized MgO·1.06Al_2_O_3_ powders containing different amounts of lithium are shown in Figure 2. The effect of lithium on the translucency of the MgO·1.06Al_2_O_3_ specimens is very apparent. In the present study, no attempts to determine optimal sintering conditions were made, with all of the compositions being sintered under the same conditions. The density, transmittance and hardness values (Table 1) of the samples prepared from near-stoichiometric powders (*n* = 1.06) all show maxima in the 0.51 at. % Li-MgO·1.06Al_2_O_3_ composition. Alumina-rich powders (*n* = 1.21) containing up to 0.53 at. % Li only reached about 95% of the theoretical density under these sintering conditions. Moreover, the samples showed no change in density, transmittance or hardness up to 0.53 at. % Li. At a higher lithium content (*i.e*., 1.04 at. %), enhanced sinterability was observed.

**Figure 3:**
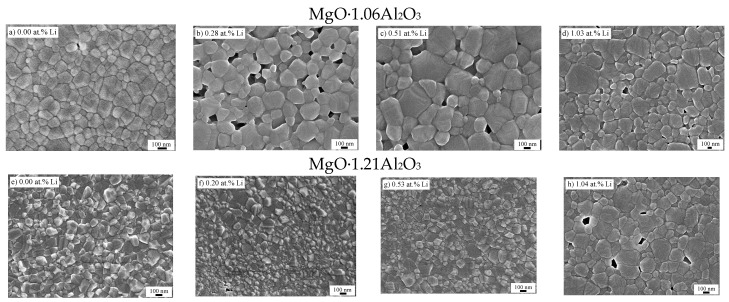
SEM micrographs of MgO·1.06Al_2_O_3_ and MgO·1.21Al_2_O_3_ SPS-processed samples. (**a**)–(**h**).

**Figure 4:**
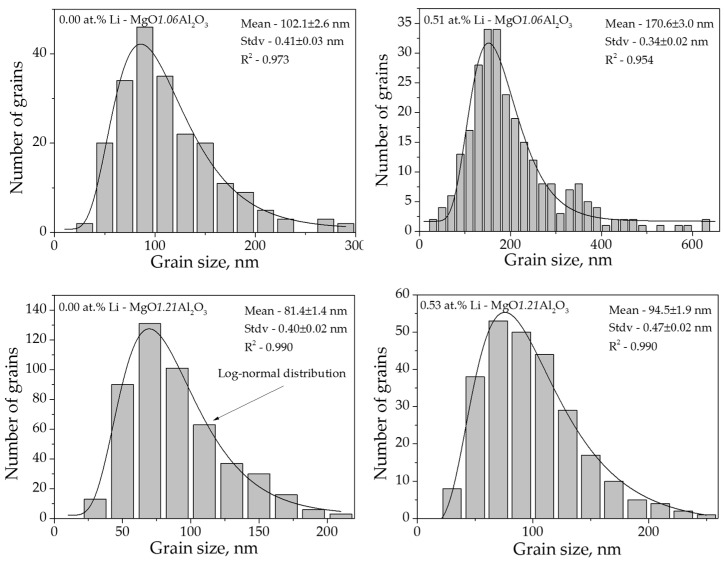
Typical grain size distribution and log-normal fitting of MgO·1.06Al_2_O_3_ and MgO·1.21Al_2_O_3_ with and without lithium addition processed by SPS.

The microstructures of the different SPS-processed specimens are presented in Figure 3. While the microstructure of the undoped MgO·1.06Al_2_O_3_ sample displayed a homogeneous nano-structure with equiaxed grains (Figure 3), the Li-doped samples consisted of two grain size populations. The doped and undoped MgO·1.21Al_2_O_3_ samples with lithium doping lower than 1.04 at. % seemed to be unaffected by the lithium addition and displayed similar equiaxed microstructures (Figure 3). The 1.04 at. % Li-MgO·1.21Al_2_O_3_ sample, however, showed a similar microstructure to the 1.03 at. % Li-MgO·1.06Al_2_O_3_ sample. The corresponding grain size distribution (an example is shown in Figure 4) exhibited a log-normal characteristic for all samples, with the calculated values summarized in Table 1. The grain size of near-stoichiometric specimens (*n* = 1.06) increased monotonically with the addition of lithium. However, this value appeared constant in alumina-rich powders (*n* = 1.21) containing up to 0.53 at. % Li. At higher lithium content (1.04 at. %), this value increased.

**Figure 5:**
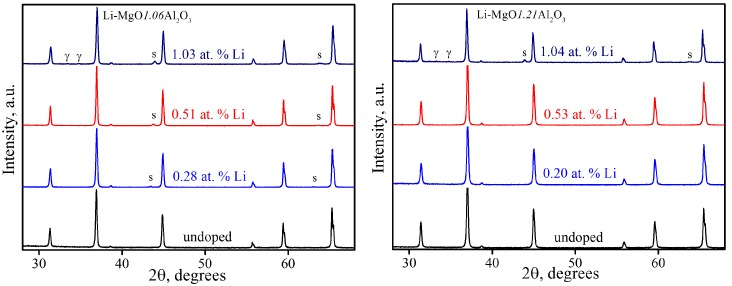
XRD patterns of Li-doped MgO·*n*Al_2_O_3_ (*n* = 1.06 and 1.21) SPS-processed samples. The precipitated MgO s.s. and γ-LiAlO_2_ phases are marked by s and γ, respectively.

The XRD patterns of the SPS-processed specimens are shown in Figure 5. The SPS-processed MgO·1.06Al_2_O_3_ and 0.00–0.51 at. % Li-MgO·1.21Al_2_O_3_ samples remained as a solid solution, while in the case of the 1.04 at. % Li-MgO·1.21Al_2_O_3_ and 0.28 through 1.03 at. % Li-MgO·1.06Al_2_O_3_ samples, Mg(Al,Li)O solid solution (MgO s.s.) and γ-LiAlO_2_ [30,31] precipitated. The amounts of MgO s.s. precipitation were calculated using the Vegard rule and data from Reference [30] and are listed in Table 1. It should be noted that the γ-LiAlO_2_ reflections were barely within the detection limit level of the XRD and were estimated to account for less than 1 wt. %. Similar behavior was found for the same powders after annealing at 1350 °C for 8 min in air [15].

In general, for samples containing up to 53.0 at. % Li, the cell parameters were 8.0810 ± 0.0005 Å and 8.0652 ± 0.0005 Å for *n* = 1.06 and 1.21, respectively. At higher Li content, both 1.03 at. % Li-MgO·1.06Al_2_O_3_ and 1.04 at. % Li-MgO·1.21Al_2_O_3_ samples displayed the same cell parameter (8.0776 ± 0.0004 Å).

### 3.2. Grain Growth

**Figure 6:**
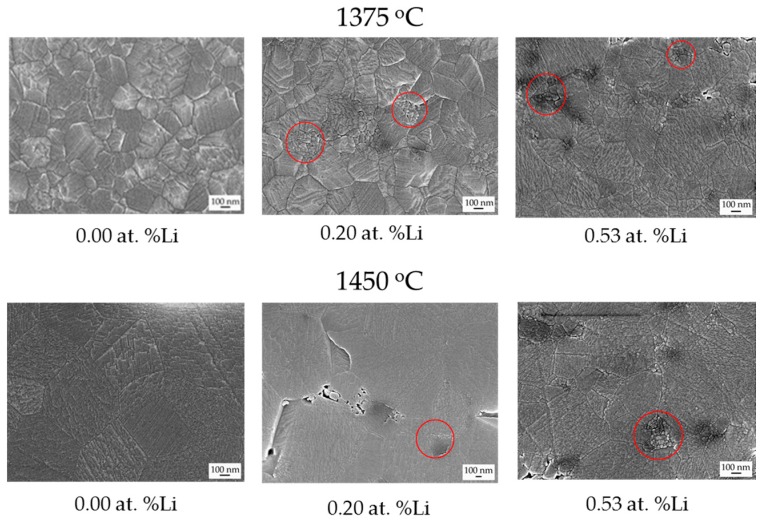
Micrographs of the undoped, 0.2 and 0.53 at. % Li-doped MgO·1.21Al_2_O_3_ samples after heat-treatment at 1375 and 1450 °C for 24 h. The presence of the fine grain clusters is marked by red circles.

The undoped, 0.28 and 0.53 at. % Li-doped MgO·1.21Al_2_O_3_ SPS-processed samples remained as a solid solution, all the while exhibiting homogeneous microstructures with equiaxed polyhedral-shaped grains. To reveal the effect of lithium on grain growth mechanisms, the grain sizes resulting from a set of heat treatments at various temperatures and times were measured (Table 2, Figure 6).

The undoped MgO·1.21Al_2_O_3_ sample showed monotonic grain growth with temperature and time. The lithium-doped samples, however, presented a more complex behavior. At low temperatures and short holding times, the lithium-doped samples showed a monotonic-like behavior similar to the undoped samples. At higher temperatures (*i.e*., 1450 °C, 8 h) or longer dwelling periods (*i.e*., 1300 °C, 24 h), the 0.53 at. % Li-MgO·1.21Al_2_O_3_ sample displayed lesser growth than the 0.20 at. % Li-MgO·1.21Al_2_O_3_ sample (Figure 6). After a longer thermal exposure, namely 72 h at 1450 °C (Figure 6), the doped samples showed enhanced grain growth, reaching a size double that of the undoped sample.

Closer examination of the SEM images of the samples after heat treatment for 24 h at 1375 and 1450 °C (Figure 6) revealed the presence of small clusters of fine grains between larger grains in the doped samples. This finding suggests that lithium-rich phases may have precipitated during the heat treatments, which could explain the growth behavior of the doped samples.

**Figure 7:**
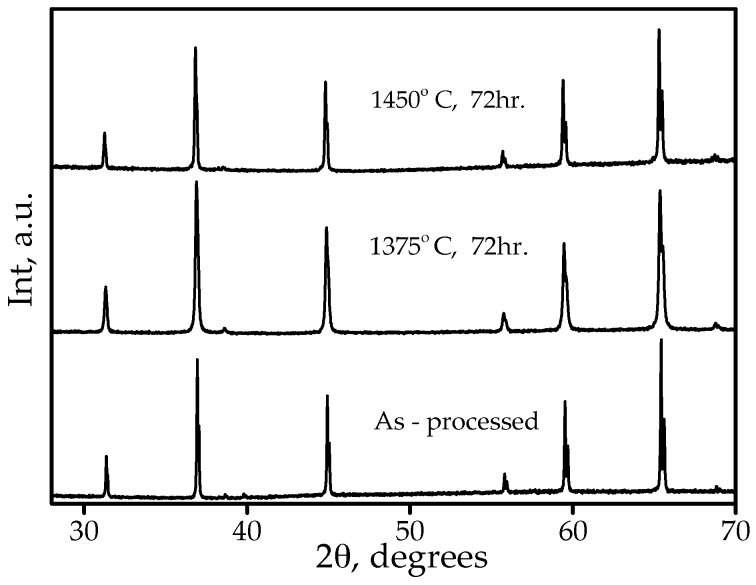
XRD spectra of as-processed and heat-treated to 1375 °C (72 h) and 1450 °C (72 h) samples.

Unfortunately, XRD analysis of these samples indicated only the presence of a spinel phase (Figure 7). Although no second phase was found, it might still be present, but it would remain undetected by the XRD technique if the phase only had a minor vol % and nano-sized dimensions [32].

**Figure 8:**
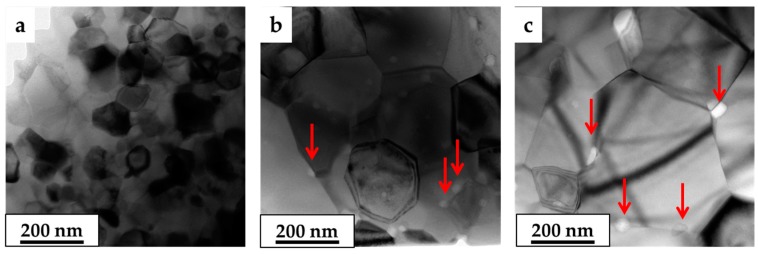
BF-TEM images of 0.20 at. % Li-MgO·1.21Al_2_O_3_ spinel system as-processed (**a**); heat-treated at 1450 °C for 24 h (**b**); and heat-treated at 1450 °C for 72 h (**c**). Second phase in grain boundary, especially in triple points, is clearly visible in the heat-treated samples.

**Figure 9:**
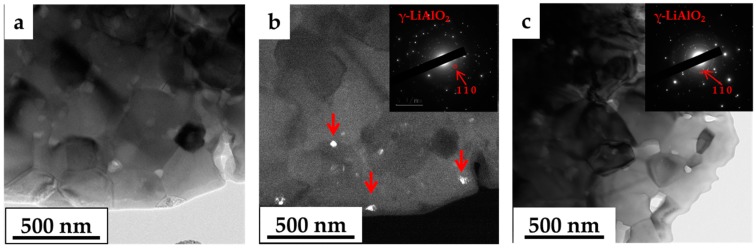
BF-TEM image of SPS spinel samples doped with 0.20 at. % Li after heat treatment at 1450 °C for 24 h (**a**); the white particles in the DF-TEM image are the γ-LiAlO_2_ phase (**b**); BF-TEM image of spinel samples doped with 0.20 at. % Li after heat treatment at 1450 °C for 72 h (**c**); the selected area diffraction patterns indicate the reflection of (1 1 0) of the γ-LiAlO_2_ phase.

To identify the nature of these fine grains, TEM analysis was performed on 0.20 at. % Li-MgO·1.21Al_2_O_3_ before and after heat treatment at 1450 °C for 24 and 72 h (Figure 8). The TEM image of the SPS-processed 0.20 at. % Li-MgO·1.21Al_2_O_3_ sample (Figure 8a) showed only spinel grains and confirmed the results of the XRD investigation regarding phase composition. After heat treatment at 1450 °C for 24 h, the presence of nano-particles of γ-lithium aluminate at the grain boundaries was detected (Figure 8b and Figure 9).

**Figure 10:**
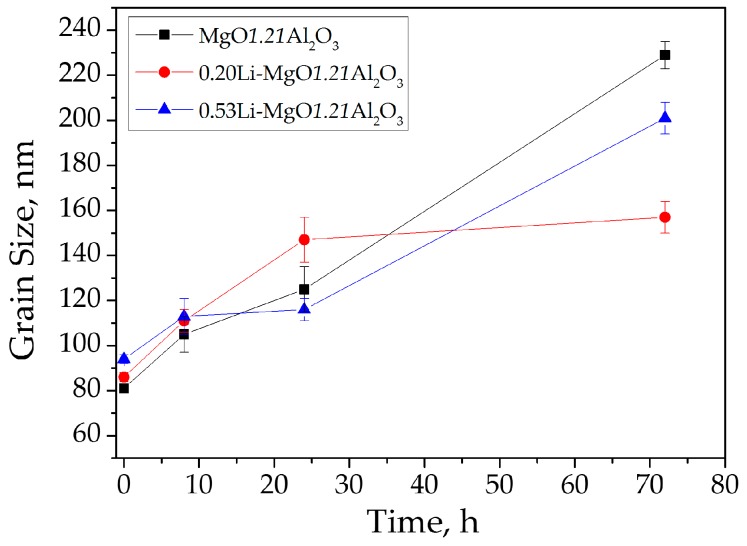
Grain sizes *vs.* annealing time of undoped, 0.2 and 0.53 at. % Li-doped MgO·1.21Al_2_O_3_ samples at 1300 °C.

In a previous study, we showed that the solubility limit of lithium in a spinel structure is controlled both by the Al-to-Mg ratio and by grain size [15]. Thus, even though no signs of second phase precipitation were present in the as-sintered 0.20 and 0.53 at. % Li-MgO·1.21Al_2_O_3_ samples, additional grain growth would promote lithium segregation to the grain boundaries and precipitation of a second phase. The segregation of lithium to the grain boundary increases the grain growth rate by reducing the grain boundary energy [15]. On the other hand, second phase precipitation impedes grain growth via the Zener pinning mechanism [33,34,35,36]. Such behavior can be seen in Figure 10. The 0.2 at. % Li-MgO·1.21Al_2_O_3_ spinel shows enhanced grain growth up to 24 h (<D> ~140 nm), after which time the growth is inhibited for a prolonged period of annealing due to second phase precipitation. In the more Li-rich samples (*i.e*., 0.53 at. % Li), grain growth was inhibited at an early stage due to earlier second phase appearance. Further coarsening was related to precipitate coarsening followed by the grain coarsening [36].

Activation energy analysis of undoped, 0.2 and 0.53 at. % Li-doped MgO·1.21Al_2_O_3_ grain growth was performed using the phenomenological kinetic grain growth equation:

$$G_{t}^{n}-G_{0}^{n}=K_{0}texp\left(-\frac{Q}{RT}\right)$$

where $G_{t}$ and $G_{0}$ are the grain sizes at times *t* and *t* = 0, respectively, *n* is the grain growth exponent, $K_{0}$ is the pre-exponential constant of the diffusion coefficient, *Q* is the activation energy for grain growth, *T* is the absolute temperature, and *R* is the gas constant.

The grain growth exponent or n value is readily determined as the inverse of the slope of a log *G* *vs.* log *t* plot. Using the original particle size as *G*_0_, the grain size data can be fitted to linear lines with similar correlation factors (*R* = 0.998 and 0.937) for both the grain growth exponents of *n* = 2 (grain boundary–controlled diffusion) and *n* = 3 (lattice-controlled diffusion). This is in agreement with other works using either *n* = 2 or 3 [37,38]. Using *n* = 2, the activation energy and kinetic constant (*K*_0_) for undoped MgO·1.21Al_2_O_3_ were found to be 288 ± 40 kJ·mol^−1^ and 2.09 × 10^6^ µm^2^/h. These values are in agreement with other data and are found between the values for MgAl_2_O_4_ and MgO·1.56Al_2_O_3_ (Table 3). The activation energies and *K*_0_ for 0.2 and 0.53 at. % Li-MgO·1.21Al_2_O_3_ were found to be 670 ± 45, 543 ± 40 kJ·mol^−1^ and 3.41 × 10^18^, 3.78 × 10^14^ µm^2^/h, respectively; these values are significantly higher than those of the undoped sample. These findings are in line with the effect of the Zener pining mechanism, where grain growth is impeded at early stages by the secondary phase. Once the secondary phase has grown and the impediment is lifted, the spinel grains show enhanced growth (see data in Table 2) that can be attributed to the effect of lithium on the diffusion, by way of imposing oxygen vacancies [9,10,11,15].

## 4. Summary

The effects of lithium doping on the sintering and grain growth kinetics of non-stoichiometric nano-MgO·*n*Al_2_O_3_ spinel with *n* = 1.06 and 1.21 were studied using a spark plasma sintering apparatus. The near-stoichiometry (*n* = 1.06) Li-doped samples showed higher sinterability in comparison with the aluminum-rich samples (*n* = 1.21) but also lower phase stability, with Mg(Li,Al)O and γ-LiAlO_2_ phases precipitating during the course of the sintering process. Still, the aluminum-rich system (*n* = 1.21) showed greater phase stability up to 1 at. % of lithium for samples with grain sizes lower than 100 nm. The grain growth study indicated that in the Li-MgO·*n*Al_2_O_3_ system, grain growth was controlled by the Zener pining mechanism, where γ-LiAlO_2_ precipitated at the grains boundaries. The activation energies of the undoped, 0.20 and 0.53 at. % Li-MgO·1.21Al_2_O_3_ samples were 288 ± 40, 670 ± 45 and 543 ± 40 kJ·mol^−1^, respectively.

## Figures and Tables

**Table 1:** Cell parameter grain size, density, transmittence, hardness and MgO s.s. amount for SPS-processed Li-doped MgO·*n*Al_2_O_3_ (*n* = 1.06 and 1.21) samples.

Li (at. %)	A (Å)	D (nm)	ρ (g/cm^3^)	Trans. (500 nm) (%)	Trans. (1000 nm) (%)	Hardness (GPa)	Mg_x_(Al,Li)_1–x_O			
							Wt. %	*a* (Å)	Composition *	
									Mg	(Al, Li)
	Li-MgO·1.06Al_2_O_3_									
-	8.0810 (1)	102 ± 3	3.49 ± 0.01	-	-	14.3 ± 0.2	-	-	-	-
0.28 ± 0.02	8.0815 (1)	160 ± 5	3.54 ± 0.01	3.5 ± 0.1	14.4 ± 0.1	14.7 ± 0.3	0.9 ± 0.1	4.180 (9)	0.86	0.14
0.51 ± 0.05	8.0784 (1)	171 ± 3	3.56 ± 0.01	25.0 ± 0.1	45.3 ± 0.1	15.3 ± 0.4	1.8 ± 0.1	4.141 (9)	0.68	0.32
1.03 ± 0.10	8.0773 (1)	150 ± 8	3.54 ± 0.01	7.4 ± 0.1	22.9 ± 0.1	14.6 ± 0.5	2.9 ± 0.2	4.127 (9)	0.63	0.37
	Li-MgO·1.21Al_2_O_3_									
-	8.0647 (1)	81 ± 1	3.48 ± 0.01	-	-	14.4 ± 0.5	-	-	-	-
0.20 ± 0.02	8.0654 (1)	86 ± 2	3.49 ± 0.01	-	-	14.2 ± 0.2	-	-	-	-
0.53 ± 0.06	8.0656 (4)	94 ± 2	3.48 ± 0.01	-	-	14.1 ± 0.4	-	-	-	-
1.04 ± 0.10	8.0779 (2)	138 ± 4	3.61 ± 0.01	2.0 ± 0.1	10 ± 0.1	13.9 ± 0.3	2.3 ± 0.3	4.117 (9)	0.6	0.4

* Calculated using the Vegard rule and data from Doman’s work [30].

**Table 2:** Grain sizes of heat-treated, undoped and 0.28 and 0.53 at. % Li-doped MgO·1.21Al_2_O_3_ samples.

Temperature (°C)/Time (h)	Grain Size (nm)		
	8	24	72
MgO·1.21Al_2_O_3_			
**1300**	105 ± 8	125 ± 10	229 ± 6
**1375**	131 ± 10	200 ± 19	272 ± 26
**1450**	181 ± 12	292 ± 26	513 ± 22
0.28-MgO·1.21Al_2_O_3_			
**1300**	111 ± 5	147 ± 10	157 ± 7
**1375**	154 ± 5	242 ± 18	249 ± 18
**1450**	306 ± 11	438 ± 9	991 ± 115
0.53-MgO·1.21Al_2_O_3_			
**1300**	113 ± 8	116 ± 5	201 ± 7
**1375**	149 ± 8	237 ± 53	280 ± 8
**1450**	197 ± 18	300 ± 6	948 ± 47

**Table 3:** Grain growth parameters for 0–0.53 at. % Li-MgO·1.21Al_2_O_3_.

MgO·*n*Al_2_O_3_	Activation Energy for Grain Growth (kJ/mol)	ln(*K*_0_)
	Undoped	
1.56 (Chiang [39])	248 ± 29	16.35
1.21 (This study)	288 ± 40	14.55
1.013 (Chiang [39])	422 ± 10	28.23
~1.00 (Bratton [40])	462	30.54
at. % Li	Lithium doped *n* = 1.21 (This study)	
0.20	670 ± 45	42.67
0.53	543 ± 40	33.56

## Abbreviations

The following abbreviations are used in this manuscript:

SPS	spark plasma sintering
HRSEM	high resolution scanning electron microscope
HRTEM	high resolution transmission electron microscope
HAADF	high angle annular dark field
STEM	scanning transmission electron microscope
XRD	X-ray powder diffraction
MgO s.s.	Mg(Al,Li)O solid solution
BF-TEM	bright field transmission electron microscope
DF-TEM	dark field transmission electron microscope
